# Translation-Targeting RiPPs and Where to Find Them

**DOI:** 10.3389/fgene.2020.00226

**Published:** 2020-03-31

**Authors:** Dmitrii Y. Travin, Dmitry Bikmetov, Konstantin Severinov

**Affiliations:** ^1^Center of Life Sciences, Skolkovo Institute of Science and Technology, Moscow, Russia; ^2^Institute of Gene Biology, Russian Academy of Sciences, Moscow, Russia; ^3^Institute of Molecular Genetics, Russian Academy of Sciences, Moscow, Russia; ^4^Center for Precision Genome Editing and Genetic Technologies for Biomedicine, Institute of Gene Biology, Russian Academy of Sciences, Moscow, Russia; ^5^Waksman Institute for Microbiology, Rutgers, Piscataway, NJ, United States

**Keywords:** RiPPs, ribosome, antibiotics, LAPs, YcaO, azol(in)e-modified peptides, genome mining

## Abstract

Prokaryotic translation is among the major targets of diverse natural products with antibacterial activity including several classes of clinically relevant antibiotics. In this review, we summarize the information about the structure, biosynthesis, and modes of action of translation inhibiting ribosomally synthesized and post-translationally modified peptides (RiPPs). Azol(in)e-containing RiPPs are known to target translation, and several new compounds inhibiting the ribosome have been characterized recently. We performed a systematic search for biosynthetic gene clusters (BGCs) of azol(in)e-containing RiPPs. This search uncovered several groups of clusters that likely direct the synthesis of novel compounds, some of which may be targeting the ribosome.

## Introduction

Antibiotics are extensively used worldwide in healthcare, agriculture, and food preservation. However, development and the spread of resistance to most antibiotics discovered during the second half of the 20th century in the course of the so-called “golden era of antibiotics” has become a global threat (Brown and Wright, 2016). With multinational pharmaceutical corporations exiting the field, the search for novel natural products, which remain a major source of novel bioactive compounds including antibiotics (Li and Vederas, 2009; Moloney, 2016), is largely concentrated in academia and small companies (Wright, 2017). Classical activity-based strain screening approaches, which are costly and which often result in a rediscovery of the already known compounds, are giving way to “smarter” techniques (Baltz, 2019). The genome mining approach relies on a “from genes to products” paradigm, which is the opposite of conventional activity-based antibiotic searches, and critically depends on the rapid accumulation of genomic data in publicly available databases (Ziemert et al., 2016).

Genome mining for novel metabolites begins with *in silico* predictions of functions of the groups of genes called “biosynthetic gene clusters” (BGCs), whose products may take part in the biosynthesis of a certain metabolite. Making specific predictions about the structure of the final compound enables better prioritizing of candidate BGCs for subsequent time-consuming downstream experimental validation. Both proteinogenic and non-proteinogenic amino acids can act as building blocks for the production of specialized metabolites, leading to a great diversity of naturally occurring bioactive peptides. Peptide natural products originating from bacteria and fungi and currently used as antibiotics are dominated by non-ribosomal peptides (NRPs), assembled by large multisubunit enzymatic complexes (Süssmuth and Mainz, 2017). With the number of known NRP BGCs steadily growing, our abilities to predict both the amino acid sequence and tailoring modifications of final compounds based on the sequences of NRP synthases and additional enzymes encoded in BGCs improve as well; however, the complete “nonribosomal code” is not yet known (Ackerley et al., 2016).

In addition to NRPs, ribosomally synthesized and post-translationally modified peptides (RiPPs) comprise another rapidly expanding class of bioactive peptides. They are produced by posttranslational modifications (PTMs) of ribosomally synthesized precursors by dedicated enzyme machinery (Arnison et al., 2013). Compared to those of NRPs, RiPP BGCs are generally smaller and contain a precursor peptide gene, which enables better prediction of the final product structure based not only on the properties of enzymes involved in biosynthesis but also on the chemical structure of the initial peptide substrate. While the number of identified RiPPs grows, there is still an enormous space for the discovery of new compounds expanding the diversity within the already known RiPP subclasses and of entirely new groups of RiPPs harboring novel modifications, as evidenced, for example, by the recently described ranthipeptides (Hudson et al., 2019) and streptide-like RiPPs (Schramma et al., 2015).

**FIGURE 1:**
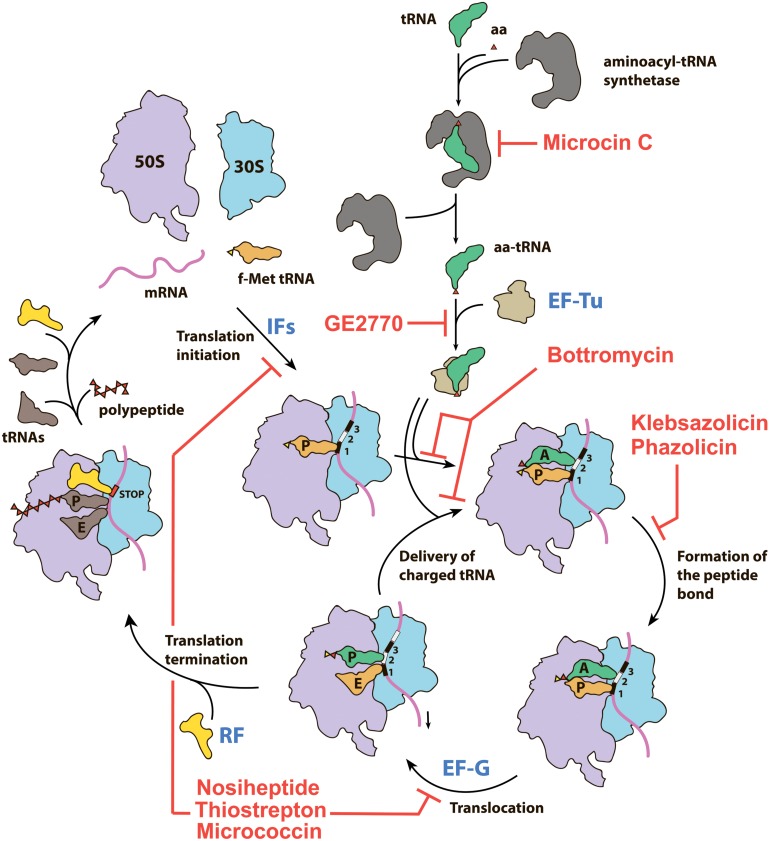
Overview of the prokaryotic translation process and its steps targeted by various RiPPs. IFs—initiation factors, RF—release factor, aa—amino acid, aa-tRNA—aminoacyl tRNA, f-Met tRNA—initiator N-formylmethionine tRNA.

Among the RiPPs exhibiting antibacterial activity, there are examples of compounds targeting various validated drug targets, including bacterial RNA polymerase (microcin J25; Delgado et al., 2001), DNA gyrase [microcin B17 (McB); Heddle et al., 2001], and the cell membrane (lanthibiotics; Chatterjee et al., 2005). The prokaryotic ribosome, another key target of many antibiotics currently in use (reviewed in Wilson, 2014; Polikanov et al., 2018), is also inhibited by certain RiPPs. In addition to RiPPs directly interacting with the ribosome, there are those that block translation by binding to elongation factors or inhibiting the activity of aminoacyl-tRNA synthetases. In this review, we briefly summarize the available data on the structure, biosynthesis, mode of action, and BGC composition of the known RiPPs inhibiting different steps of translation (Figure 1). In addition, we specifically explore the genomic landscape of azol(in)e-containing RiPPs in an attempt to predict novel RiPPs inhibiting translation. Although definitive predictions of the compound mode of action based entirely on the genomic data can be made only in rare cases (see, for example, the self-resistance guided identification of new topoisomerase inhibitors; Panter et al., 2018), the results of our search define new subclasses of azol(in)e-containing RiPPs, which may include the translation inhibitors.

### Thiopeptides

**FIGURE 2:**
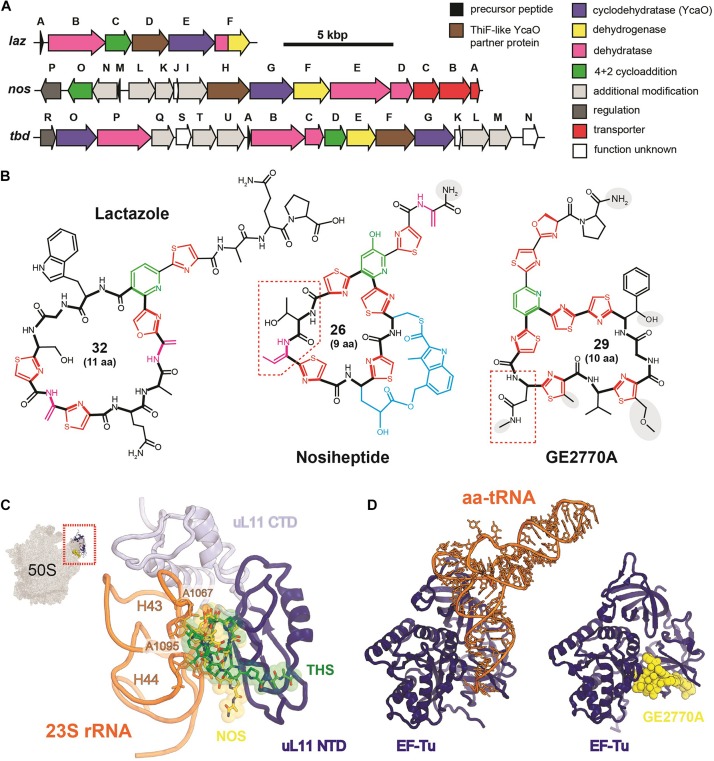
Thiopeptides. **(A)** Biosynthetic gene clusters of lactazole (a minimal thiopeptide-encoding BGC), nosiheptide, and GE2270A. Functions of encoded proteins are listed on the right. **(B)** Chemical structures of lactazole, nosiheptide, and GE2270A. Azol(in)e cycles are shown in red, six-membered central azacycles in green, dehydrated amino acids in pink, and methylindole acid-containing second ring system of nosiheptide in blue. Auxiliary tailoring modifications are highlighted with gray background. The number of atoms and amino acid residues in the macrocyclic system is indicated for each compound. Macrocycles are shown in bold. Red dashed polygons show conserved residues characteristic for ribosome targeting (nosiheptide) and EF-Tu targeting (GE2270A) thiopeptides. **(C)** Mode of nosiheptide and thiostrepton interaction with the ribosome (PDB IDs 2ZJP and 3CF5; Harms et al., 2008). Nosiheptide is yellow, thiostrepton is green, uL11 ribosomal protein CTD (C-terminal domain) is lightblue, NTD (N-terminal domain)—blue, H43 and H44 helices of 23S rRNA are orange, residues A1067 and A1095 (*E. coli* nomenculature), involved in the rRNA-antibiotic interaction are shown as sticks. **(D)** Mode of action of GE2770A. Elongation factor EF-Tu (blue) is shown in complex with aminoacyl tRNA (aa-tRNA, orange) and GE2270A (yellow) (PDB IDs 1B23 and 2C77, respectively, Nissen et al., 1999; Parmeggiani et al., 2006). The binding of GE2270A prevents the interaction of EF-Tu with aa-tRNA acceptor stem.

Thiopeptides comprise one of the best-studied subclasses of RiPPs, with more than 100 compounds characterized to date. Produced predominantly by *Actinobacteria*, they demonstrate various activities including antibacterial and antiplasmoidal (Gaillard et al., 2016), which result from their ability to inhibit translation by the prokaryotic ribosome and the ribosome within the apicoplast of the malaria parasite *Plasmodium falciparum* (Clough et al., 1997). All known thiopeptides have the specific set of biosynthetic genes in their BGCs (Figure 2A), share common structural features (Figure 2B), and use two major mechanisms for translation inhibition: they either interact directly with the ribosome (Figure 2C) or prevent the binding of aminoacyl-tRNA by the elongation factor EF-Tu (Figure 2D).

The first group of thiopeptides is characterized by the small size of a macrocycle (26 atoms) and a conserved region (Figure 2B, nosiheptide, red dashed frame) essential for the interaction with their binding site on the large ribosome subunit, GTP-ase associated center (GAC). Thiostrepton, nosiheptide, and micrococcin, all belonging to this group, were co-crystallized with the large ribosome subunit of *Deinococcus radiodurans*. While most ribosome-targeting antibiotics contact only rRNA, these thiopeptides interact with both rRNA and ribosomal proteins, binding in a cleft formed by the N-terminal domain of the ribosomal protein uL11 and the loops of helices H43 and H44 of the 23S rRNA (Figure 2C; Harms et al., 2008). Their binding site overlaps with the binding sites of IF2, EF-G, and EF-Tu. Consistently, thiopeptides inhibit initiation (Brandi et al., 2004), translocation (Rodnina et al., 1999), and tRNA delivery to the ribosome (Gonzalez et al., 2007). The practical applications of naturally occurring thiopeptides are limited by their poor water solubility (Just-Baringo et al., 2014). Nevertheless, nosiheptide is used as a growth stimulating additive in mixed animal food, while thiostrepton is used to treat skin infections in animals.

GE2270A, isolated in the early 1990s from actinobacterium *Planobispora rosea* (Selva et al., 1991), is a representative of the second functional group of thiopeptides. It binds to the elongation factor EF-Tu in a complex with GTP and prevents the formation of the ternary complex with aminoacyl-tRNA (Figure 2D; Heffron and Jurnak, 2000). A derivative of GE2770A with an altered C-terminus named LFF571, proved to be effective and safe in phase II clinical trials against *Clostridium difficile* infections, a rare case when a RiPP-inspired molecule reached clinical trials (Jarrad et al., 2015). The GE2270A binding site is located between EF-Tu domains I and III and partially overlaps with the binding site of polyketide antibiotic pulvomycin, an interesting example of two chemically unrelated compounds adopting a similar mode of inhibition of the same molecular target (Parmeggiani et al., 2006). Other thiopeptides demonstrating similar modes of action, e.g., thiomuracin and GE37468A, are all characterized by the medium size of the macrocycle (29 atoms) and the presence of a conserved Asn or MeAsn residue (Figure 2B, GE2770A, red dashed frame), required for the interaction with EF-Tu (Young et al., 2012).

The “core” set of PTMs characterizing most thiopeptides includes the installation of azol(in)e heterocycles, dehydration of amino acids, and macrocyclization *via* the formation of a six-membered azacycle. Azole cycles (Figure 2B, red) are synthesized in a two-step reaction from amino acids with a nucleophilic group in their side chains. First, an YcaO-domain cyclodehydratase together with ThiF-like partner protein (the latter is required for precursor recognition) converts Cys residues into thiazolines and Ser or Thr residues into oxazolines or methyloxazolines, respectively. Flavin mononucleotide (FMN)-dependent dehydrogenase can further oxidize azoline cycles into aromatic azoles. The mechanism of azole installation and diversity of azol(in)e-containing RiPPs were recently reviewed by Burkhart et al. (2017).

The formation of dehydrated amino acids (dehydroalanine from Ser residues and dehydrobutyrine from Thr, Figure 2B, pink) in thiopeptides proceeds *via* a glutamylation-elimination mechanism with tRNA^Glu^ functioning as a donor of glutamyl (Hudson et al., 2015). The enzymes catalyzing this reaction are encoded by two separate genes (so-called “split” LanB), which are also found in BGCs of other, unrelated, RiPPs including class I lanthipeptides. The mechanisms and enzymology of dehydration of amino acids were reviewed by Repka et al. (2017). Amino acid dehydration is a prerequisite for the most remarkable modification of thiopeptides—formation of the central nitrogen-containing heterocycle (Figure 2B, green). This reaction follows the [4+2] cycloaddition mechanism (aza-Diels-Alder reaction) that is in most cases accompanied by the removal of the leader peptide and leads to the formation of a macrocycle system. The enzymes responsible for catalysis of these reactions in various biosynthetic pathways were reviewed by Jeon et al. (2017). The structure of the central azacycle is the basis for thiopeptide classification into series (from *a* to *e*) (Bagley et al., 2005).

The genes encoding the enzymes responsible for “core” PTMs mentioned above, together with a precursor peptide gene, constitute the simplest variant of thiopeptide BGC, i.e., the *laz*-cluster of lactazole synthesis (Figure 2A, Hayashi et al., 2014). Most thiopeptide BGCs are larger and encode the enzymes catalyzing additional modifications (Figure 2A, *nos-* and *tbd*-clusters) as well as transporters and regulatory proteins. The tailoring modifications may include the formation of a side ring system *via* the addition of indole derivates (Figure 2B, blue), modifications of the C-terminus to prevent hydrolysis by carboxypeptidases, glycosylation, hydroxylation, etc. The diversity and mechanisms of thiopeptide PTMs were reviewed in detail by Zheng et al. (2017).

### Linear Azol(in)e-Containing Peptides (LAPs)

The name “linear azol(in)e-containing peptides (LAPs)” refers to the only two characteristics shared by compounds from this diverse subgroup of RiPPs: they (*i*) have azol(in)e cycles installed along the polypeptide backbone and (*ii*) do not undergo macrocyclization (Arnison et al., 2013). Thus, a minimal LAP BGC comprises only a gene encoding the precursor peptide (gene A) and gene(s) coding for the enzymes involved in the installation of azole cycles: a YcaO-cyclodehydratase (the product of the D gene), which in most cases has a partner protein that is required for leader peptide recognition (either an E1-like protein, the product of the C gene, or a ThiF-like protein encoded by the F gene), and a dehydrogenase (the product of the B gene) which oxidizes azolines to azoles. In some BGCs, genes coding for C and D proteins, are fused and code for a single polypeptide (Burkhart et al., 2017). The list of additional modifications of LAPs is diverse and includes, among others, N-methylation (plantazolicin, Lee et al., 2013), the formation of dehydroamino acids and N-terminal acetylation (goadsporin, Ozaki et al., 2016), N-terminal oxyme formation, and C-terminal O-methylation (azolemycin, Liu et al., 2016).

As the set of chemical characteristics required to attribute a compound to LAPs is not particularly restrictive, the group includes compounds without any obvious sequence similarity of peptide precursors. The relationships between the known LAPs (to date there are less than two dozens of well-characterized compounds) resemble a “sea with islands,” where each “island” is formed by a group of the closely related homologs (e.g., streptolysin S with its relatives; Molloy et al., 2011) without links between the “islands.”

**FIGURE 3:**
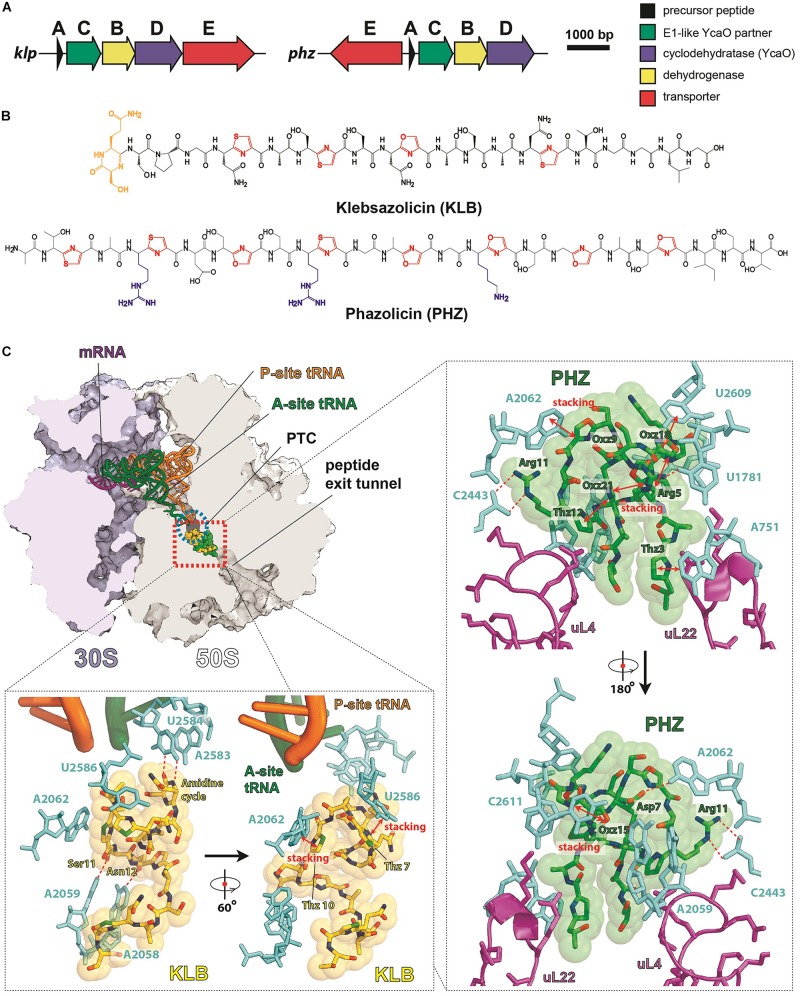
LAPs targeting the ribosome exit tunnel. **(A)** BGCs of klebsazolicin (*klpACBDE*) and phazolicin (*phzEACBD*). The functions of encoded proteins are listed on the right. **(B)** Chemical structures of klebsazolicin (KLB) and phazolicin (PHZ), azole cycles are shown in red, positively charged amino acid side chains of PHZ—in blue, the N-terminal amidine cycle of KLB is yellow. **(C)** Mechanism of translation inhibition by KLB (PDB ID 5W4K, Metelev et al., 2017) and PHZ (PDB ID 6U48, Travin et al., 2019). Interactions of the two antibiotics with the ribosome are shown: π–π stacking is denoted by red arrows and hydrogen bonds with red dashed lines. Nucleobases of 23S rRNA are cyan, ribosomal proteins are magenta, and tRNAs are orange and green. PTC—peptidyl-transferase center.

Klebsazolicin (KLB) is the first characterized translation-targeting LAP (Metelev et al., 2017). Its BGC was found in the genome of *Klebsiella pneumonia* sub. *ozaenae* and contains a gene for the precursor peptide (*klpA*), genes encoding the enzymes required for azole cycle installation (*klpCBD*), and an exporter pump gene (*klpE*) (Figure 3A). In addition to three thiazoles and one oxazole cycle, KLB has an N-terminal amidine cycle formed by the first two residues of the core part of the precursor peptide (Ser1 and Gln2; Figure 3B), a modification unique among the known LAPs. *In vitro* studies have demonstrated that this cycle is formed after the proteolytic cleavage of the leader peptide and strictly requires the YcaO-domain KlpD cyclodehydratase (Travin et al., 2018). The amidine cycle is required for KLB to function since the derivatives with a full set of azole cycles, but lacking the amidine cycle, do not inhibit translation. As it is typical for other LAPs, KLB is a narrow spectrum antibiotic: it is active against the genera closely related to *Klebsiella*, including *Yersinia* and *Escherichia*.

Cocrystallization of KLB with the *Thermus thermophilus* ribosome (which because of ease of crystallization is widely used for structural studies of ribosome-targeting compounds) revealed the molecular details of its mode of action. KLB binds in the upper part of the peptide exit tunnel in a site adjacent to the peptidyl-transferase center (PTC) (Figure 3C). Acting as a cork in the bottle, KLB blocks the passage of the nascent peptide, only allowing the synthesis of di- or tripeptides that remain associated with tRNA and stay bound to the elongating ribosome.

Phazolicin (PHZ) is another recently discovered ribosome-targeting LAP produced by soil bacterium *Rhizobium* sp. Pop5, a symbiont of wild beans *Phaseolus vulgaris* (Travin et al., 2019). In terms of the overall composition, PHZ BGC is identical to that of KLB (Figure 3A). PHZ is a 27-amino acid long peptide, every third amino acid of which is converted into an azole cycle. Unlike KLB, no modifications other than Cys and Ser side chain cyclizations are present in PHZ (Figure 3B). PHZ is active against various rhizobia that are closely related to the producing strain. Similarly, to KLB, PHZ targets the ribosome exit tunnel but does this through a different set of interactions, which were revealed by cryo-EM of the *Escherichia coli* ribosome complex with PHZ (Figure 3C). Four azole cycles of PHZ form a π–π stacking system, which stabilizes 3D globular structure of the peptide, while the three azoles are involved in stacking with nucleobases of the 23S rRNA. Unlike KLB, PHZ has three positively charged residues involved in the interactions with phosphates and other polar groups of 23S rRNA. PHZ also interacts with the loop regions of two ribosomal proteins (uL4 and uL22). Amino acid sequences of these loops confer the species-specific mode of translation inhibition by PHZ, which, unlike KLB, does not bind to *T. thermophilus* ribosome.

### Bottromycins

Bottromycins are extensively modified RiPPs that exhibit potent antimicrobial activity against the drug-resistant human pathogens including vancomycin-resistant *Enterococcus* (VRE) and methicillin-resistant *Staphylococcus aureus* (MRSA) (Shimamura et al., 2009). In early works, bottromycin A2 was demonstrated to inhibit protein synthesis both *in vitro* and *in vivo* (Tanaka et al., 1966). Further studies showed that the action of bottromycin does not interfere with the peptide bond formation and translocation steps. Bottromycins are believed to bind in the A-site of the ribosome (Otaka and Kaji, 1976, 1981, 1983) and block the interaction of aminoacyl-tRNAs with the ribosome, almost an unexploited target among the currently used antibiotics. However, further structural studies of bottromycin mechanism of action are needed to establish the details of this interaction at a molecular level, as previous studies used indirect approaches sometimes leading to contradictory conclusions.

**FIGURE 4:**
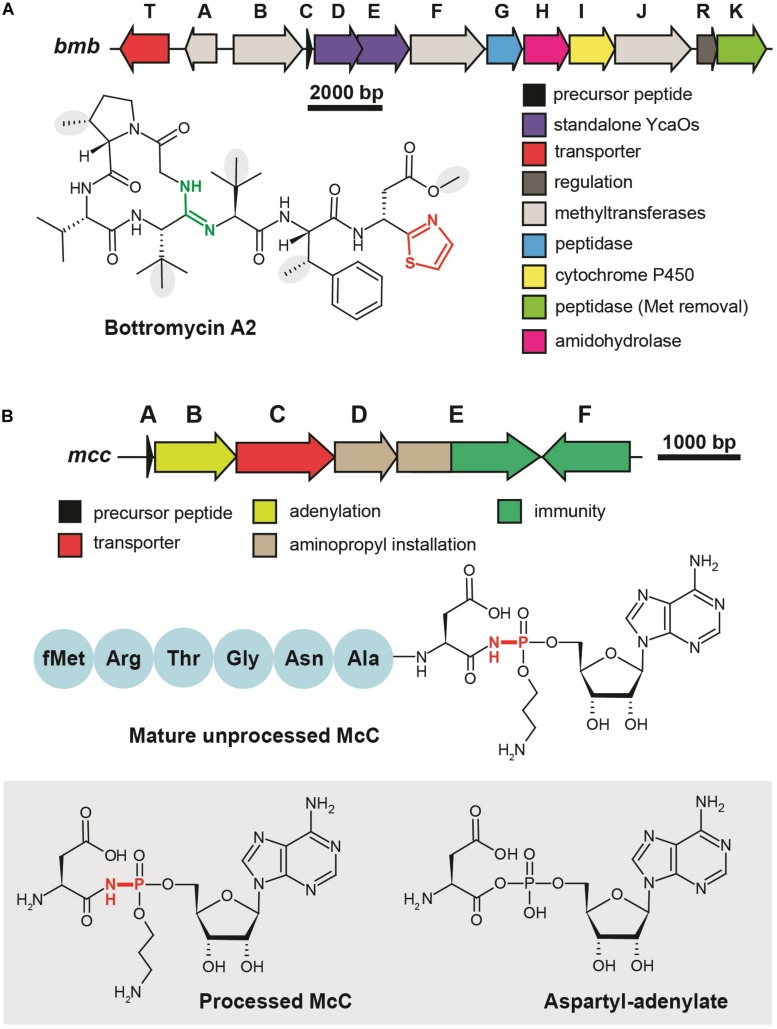
Translation-targeting RiPPs without structural information on target binding **(A)** BGC and chemical structure of bottromycin A2 from *Streptomyces bottropensis*. C-terminal thiazole is shown in red, macroamidine bond is green, and C_β_-methyl groups are shown on the gray background. **(B)** BGC of microcin C, chemical structures of unprocessed microcin C and of processed form, an analog of aspartyl adenylate. The non-hydrolyzable N–P bond is shown in red.

Although the first representative of the bottromycin family of RiPPs was isolated from *Streptomyces bottropensis* in 1957 (Waisvisz et al., 1957), more than 50 years passed until the structure of the compound was finally confirmed by total chemical synthesis (Shimamura et al., 2009). Bottromycins are eight-amino acid long extensively modified peptides originating from the N-terminal part of a precursor peptide (thus bottromycin precursor has a “follower” peptide to which modification machinery binds, rather than N-terminal “leader” common among other RiPPs). The biosynthesis of bottromycin includes many steps and was intensively studied using the untargeted methabolomics approach (Crone et al., 2016) and *in vitro* reconstitution of separate modification reactions. The PTMs characteristic to bottromycins include the formation of the N-terminal macroamidine cycle (Figure 4A, green) and C-terminal thiazole (Figure 4A, red) catalyzed by two divergent YcaO-domain enzymes acting without any partner proteins (so-called “standalone YcaOs”) (Franz et al., 2017; Schwalen et al., 2017). In addition to these cyclizations, C_β_-methylations of Pro, Phe, and Val residues, as well as O-methylation of aspartate take place (Figure 4A, gray background; Huo et al., 2012). Different methylation profiles lead to multiple forms of bottromycins produced by the same strain (Eyles et al., 2018). In addition to the genes encoding YcaO heterocyclases and methyltransferases, bottromycin BGC includes genes encoding an enzyme, which removes the N-terminal methionine residue (Mann et al., 2016), an amidohydrolase required for the follower peptide removal (Sikandar et al., 2019), a cytochrome performing oxidative decarboxylation of the C-terminal azoline into azole, and a transporter (Figure 4A).

### Microcin C and Related Compounds

Microcin C (McC) is a peptide-nucleotide antibiotic produced by *E. coli* strains bearing a plasmid with a six-gene *mcc* gene cluster (Figure 4B), which encodes a seven amino acid-long precursor peptide (MccA, MRTGNAN), enzymes responsible for its PTM (MccB, MccD, and MccE), an exporter pump (MccC), and a peptidase providing autoimmunity (MccF). The product of the *mccA* gene is adenylated by MccB, which leads to the formation of a non-hydrolyzable N-P bond between C-terminal asparagine and phosphate (Roush et al., 2008). MccD and MccE are required for additional decoration of the molecule with aminopropyl group attached to the phosphate (Kulikovsky et al., 2014). Recent studies increased the number of McC-related compounds: RiPPs of this family undergoing cytidylation instead of adenylation were discovered, and carboxymethylation of the cytidine was shown to be an additional tailoring step required for an optimal bioactivity (Serebryakova et al., 2016; Tsibulskaya et al., 2017).

Microcin C is a Trojan-horse antibiotic imported into sensitive cells *via* the inner membrane transporter YejABEF, which recognizes the peptide part of McC (Novikova et al., 2007). The McC molecule itself is not toxic for the cell; the peptide part has to be deformylated and subsequently degraded by non-specific cellular oligopeptidases (Kazakov et al., 2008) to release a nonhydrolyzable analog of aspartyl adenylate, a potent inhibitor of aspartyl-tRNA synthetase (Figure 4B, gray background) (Metlitskaya et al., 2006). This leads to the accumulation of uncharged tRNA^Asp^, inhibition of protein synthesis, and the cessation of cell growth. Thus, McC is another example of a RiPP (together with GE2270A discussed earlier), which does not directly interact with the ribosome but blocks translation by inhibiting the supply of substrates required for protein synthesis. Although McC has been studied for more than 30 years and structures of multiple enzymes involved in its biosynthesis and immunity have been determined (Agarwal et al., 2011, 2012; Dong et al., 2019), we still lack structural information about the details of McC interaction with aspartyl-tRNA synthetase.

## Genome Mining for Novel Translation Inhibiting Ripps

In light of the data discussed above, it is evident that many translation-targeting RiPPs contain azol(in)e cycles. At least in several cases where the mode of interaction with a ribosome is known, these cycles take part in stacking interactions with rRNA nucleobases thus mediating binding of the inhibitor to the target. We decided to perform a search for novel groups of azol(in)e-containing RiPPs in the genomes present in publicly available databases with a goal of identifying the putative translation inhibitors as well as other bioactive molecules. Due to their essential role in azol(in)e-containing RiPP biosynthesis, the genes encoding the YcaO-domain-containing enzymes were chosen as a starting point for our search.

In less than 10 years YcaO-domain containing enzymes went from being DUFs (domains of unknown function) to one of the most studied groups of RiPP modification proteins (Burkhart et al., 2017). It was demonstrated that YcaO enzymes play the key role in the catalysis of three distinct reactions of PTM of proteins and peptides including the installation of azoline cycles (Dunbar et al., 2012), amidines (Burkhart et al., 2017; Franz et al., 2017; Travin et al., 2018), and thioamides (Mahanta et al., 2018; Schwalen et al., 2018). A common mechanism involving the nucleophilic attack on the amide bond containing substrate with a subsequent ATP-dependent phosphorylation of the intermediate followed by phosphate elimination underlies all these activities. Three groups of proteins are regarded as YcaO partners, allowing for the interaction of the enzyme with its substrate (the recognition of the leader peptide in case of RiPP biosynthesis). These are E1-like proteins and ThiF-like proteins, fused or clustered together with azoline-forming YcaOs (Burkhart et al., 2017), and TfuA-like proteins considered to be a hallmark of the BGCs of thioamidated compounds (Santos-Aberturas et al., 2019).

**FIGURE 5:**
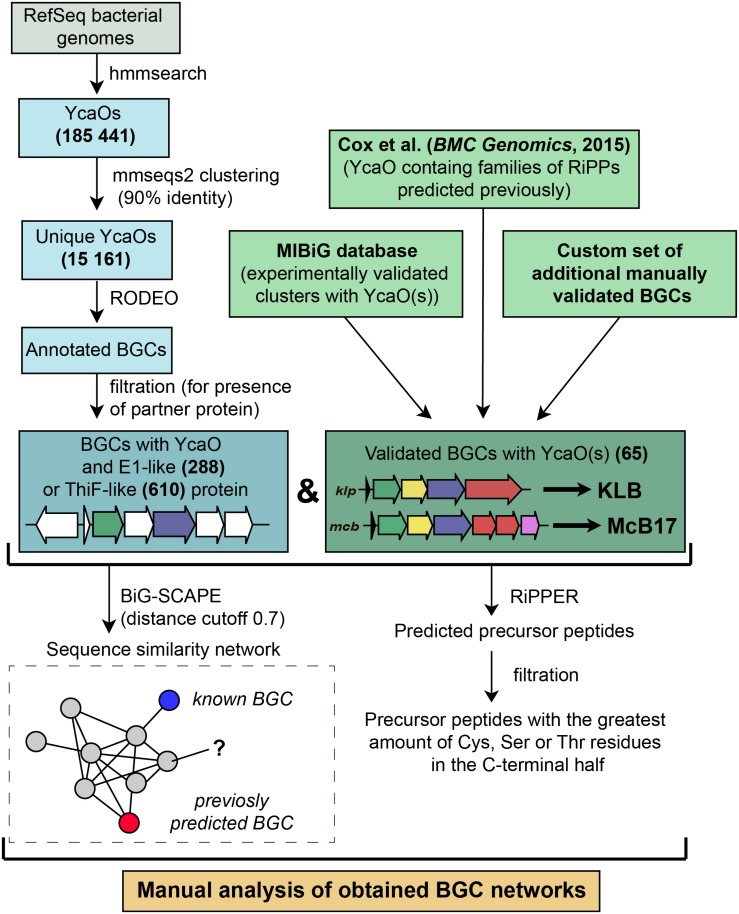
A workflow for identification of YcaO-containing RiPP BGCs.

To identify new BGCs of azol(in)e-containing compounds, we started with a sensitive search for sequences of YcaO domain-containing enzymes present in genomes from the RefSeq database (O’Leary et al., 2016). In brief, the subsequent steps included filtering, clusterization, and annotation of genomic regions surrounding the recovered *ycaO* genes (Figure 5). To visualize the obtained diversity and to identify families of BGCs, we constructed a sequence similarity network of all YcaO-containing BGCs, which was then analyzed manually (for detailed description of procedures, see section “Methods”). A curated set of characterized YcaO-containing BGCs including those present in the MIBiG database (Kautsar et al., 2019) or described elsewhere in the literature (including previous bioinformatic predictions) was used as a reference (Supplementary Table S1). In the current study, we focused only on clusters containing E1-like or a ThiF-like partner proteins and did not consider TfuA-containing BGCs or BGCs with standalone YcaOs. We also did not consider BGCs of thiopeptides and closely related RiPPs (defined as clusters containing *lanB*-like genes) as they were recently searched with various tools (Li et al., 2012; Schwalen et al., 2018). The genomic landscape of all azol(in)e-containing peptides was studied by Cox et al. (2015), however, since the time of this publication new azol(in)e-containing RiPPs with characterized modes of action (including ribosome-targeting KLB and PHZ) have been discovered, and many more sequenced genomes have been deposited in publicly available databases. Moreover, several improved methods and software have become available.

**FIGURE 6:**
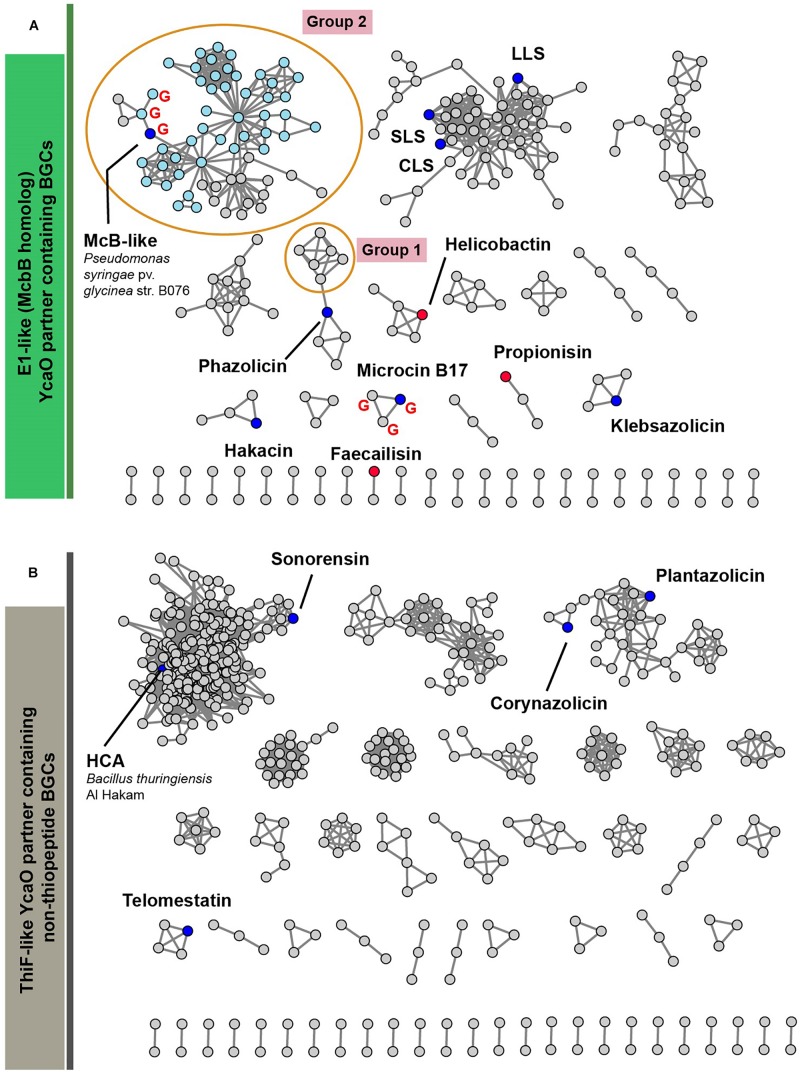
A similarity network of YcaO-containing BGCs with E1-like **(A)** and ThiF-like **(B)** partner proteins. Nodes representing BGCs of already characterized compounds are shown in blue, nodes representing BGCs analyzed in Cox et al. (2015) are red. BGCs containing an *mcbG*-homolog are denoted with red “G.” Groups of clusters discussed in the text are shown in orange ellipses. Light blue color of nodes in group 2 shows BGCs from genus *Pseudomonas.* SLS—strepolysin S, LLS—listeriolysin S, CLS—clostridiolysin S, HCA—heterocycloanthracin.

Figure 6 represents a similarity network of YcaO-containing BGCs encoding E1-like (Figure 6A) or ThiF-like (Figure 6B) YcaO partner proteins (see Supplementary Table S2 for the list of all BGCs). BGCs of the already characterized compounds from the curated dataset are shown as blue circles. In the network with E1-like partners, these include BGCs of bioactive antibacterials McB, KLB, and PHZ as well as a number of streptolysin S-like RiPPs (clostridilysin S; listeriolysin S; Cotter et al., 2008; Gonzalez et al., 2010) and hakacin, whose biosynthesis was studied *in vitro*, but the structure of the naturally produced compound remains unknown (Melby et al., 2012; Dunbar and Mitchell, 2013). In the network of ThiF-like protein containing BGCs we observed a large group of heterocycloanthracin (HCA) BGCs, which include the already characterized sonorensin (Chopra et al., 2014) and HCA from *Bacillus thuringiensis* Al Hakam (Dunbar et al., 2015).

Below, we discuss three groups of BGCs, which attracted our attention during the analysis of the networks and putative peptides encoded by these BGCs as predicted by RiPPER (Santos-Aberturas et al., 2019) in Figure 6. We consider it likely that the first group of these BGCs encodes new translation targeting RiPPs; the second may also do so, while the third was so interesting in terms of RiPP encoding clusters’ evolution, that we could not help but discuss it in this article.

### Lactazolicins

**FIGURE 7:**
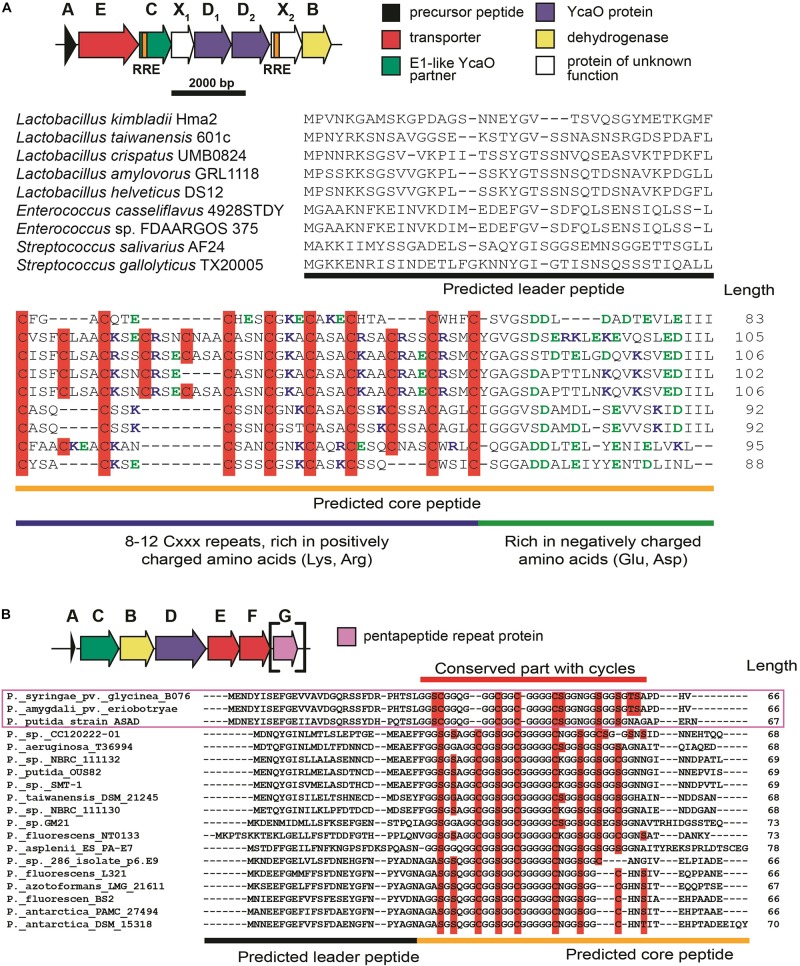
**(A)** Common BGC composition conserved among lactazolicin BGCs, proposed functions of the encoded proteins are listed on the right. RiPP recognition elements (RREs) in C and X_2_ genes are depicted. Alignment of precursor peptides of lactazolicins, predicted leader and core parts are shown. Cysteines in the core part are shown on red background, positively charged amino acids of the predicted core are blue, and negatively charged residues are green. **(B)** The composition of McB-like BGC from pseudomonads. *mcbG* homolog is shown in square brackets as it is not present in the majority of clusters from pseudomonads, the genes are colored according to the color scheme in **(A)**. Alignment of precursor peptides predicted with RiPPER, those encoded in PRP gene-containing BGCs are in magenta frame. Potentially cyclized residues are shown with red background, predicted core and leader parts are shown.

The first group of BGCs contains clusters from the representatives of the genus *Lactobacillus*, which form a connected component with PHZ BGC (Figure 6, Group 1). Analysis of these BGCs and their homologs from genera *Enterococcus* and *Streptococcus* found with an additional BLAST search revealed that all these BGCs share the same set of genes, which, in addition to modification machinery and export pump homologs of those in PHZ BGC (Figure 7A, genes E, C, B, and D_2_), includes three auxiliary genes (Figure 7A, genes X_1_, D_1_, and X_2_). The product of gene D_1_ is the second YcaO protein. It is distinct from the product of the D_2_ gene and lacks the C-terminal PxP-motif, found in azoline-forming YcaOs and involved in catalysis (Ghilarov et al., 2019). According to the results of HHPred (Söding et al., 2005), the product of gene X_2_ is distantly related to ThiF/MccB/PaaA proteins and contains a RiPP recognition element (RRE) — a domain found in different RiPP modification enzymes binding leader peptides (Burkhart et al., 2015). The presence of the second YcaO and of the X_2_ gene product, which could function either as a partner protein or an independent adenylating enzyme (Ghodge et al., 2016; Dong et al., 2019), makes additional modifications of the precursor peptide highly probable. We were unable to detect any homologs of the X_1_ gene product among the known proteins.

Following the conventional practice of giving names to the proposed new groups of compounds (Cox et al., 2015) and in accordance with the nomenclature recommended for LAPs (Arnison et al., 2013), we named this group of putative translation inhibitors *lactazolicins*. All lactazolicin clusters encode 83-106 amino acid-long putative precursor peptides with 8–12 repeats of the [Cxxx] motif in the N-terminal part of the predicted core segment (Figure 7A). HCAs represent an already known group of RiPPs, which have a similar pattern of repeated cysteine residues in the core part (Haft, 2009). However, HCA precursors (also found in our search, Figure 6B, the largest group of BGCs) have the [Cxx] motif repeated rather than [Cxxx], and the overall composition of HCA BGCs also differs significantly from that of lactazolicin BGCs. Unlike HCAs, where the [Cxx]-repeat containing part of the precursor is rich in glycines, the N-terminal [Cxxx] repeat-containing part of lactazolicin precursors is enriched in positively charged amino acids (Arg, Lys). In the cases of PHZ and proline-rich peptides (which do not belong to RiPPs but also target the ribosome exit tunnel) (Gagnon et al., 2016), the side chains of positively charged amino acids take part in the interaction with phosphate groups of rRNA. We thus hypothesize that lactazolicins may also affect translation.

### Microcin B17-Like BGCs From Pseudomonads

Microcin B17 is a DNA-gyrase-targeting LAP produced by some strains of *E. coli*. The McB BGC contains a set of enzymes similar to those encoded by the KLB and PHZ BGCs and an additional gene *mcbG*, which encodes a pentapeptide repeat protein (PRP) (Li et al., 1996; Heddle et al., 2001). McbG is likely a DNA mimic that decreases the formation of toxic gyrase-DNA complexes trapped by McB, thus protecting the gyrase in the McB-producing cell (Hegde et al., 2005; Vetting et al., 2011). Clusters similar to that of McB were described in the genomes of several pathovars of *Pseudomonas syringae* and their products also target gyrase (Metelev et al., 2013).

A relatively large network of clusters retrieved by our search (Figure 6A, Group 2) contains no previously characterized representatives except for an *mcb-*operon homolog from *P. syringae* (blue circle). However, several of these clusters (marked with red letter G) contain a gene coding for a PRP protein. The overall sequence similarity and the distribution of potentially cyclizable residues in precursor peptides from clusters with and without the PRP gene differ (Figure 7B). Thus, it is highly probable that *mcb*-like clusters without a PRP gene encode a RiPP with a target distinct from DNA gyrase. While we cannot establish whether these are translation-targeting RiPPs, compounds with the same set of proteins in their BGC (KLB and PHZ) do affect translation.

### Flavazolicins

**FIGURE 8:**
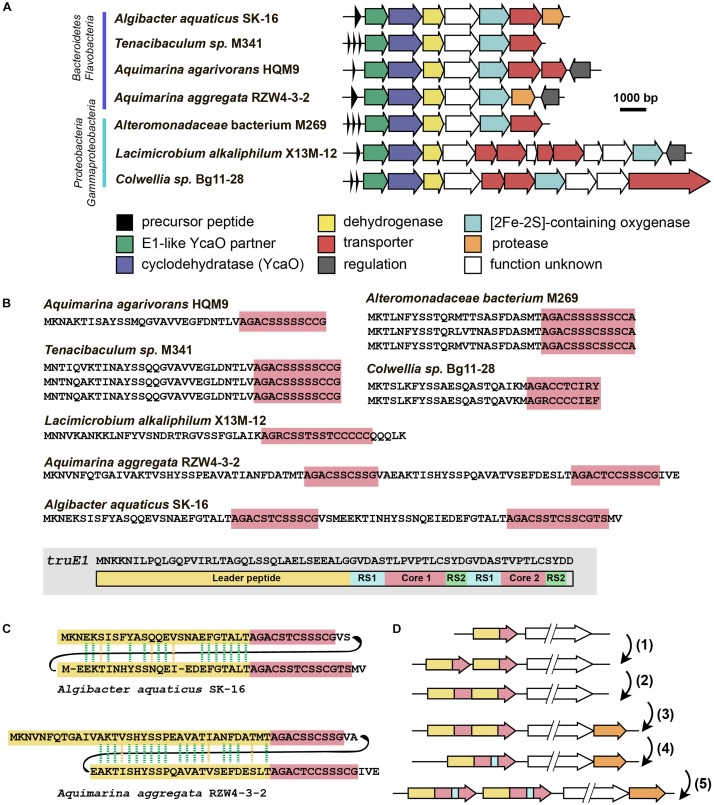
Flavazolicines. **(A)** Comparison of biosynthetic gene clusters encoding a putative new group of LAPs found in *Flavobacteria* and *Gammaproteobacteria* genomes. Predicted functions of the Encoded proteins are listed below. **(B)** Comparison of precursor peptides of flavazolicins. Conserved core sequences containing cyclizable residues are shown with red background. The precursor peptide sequence of cyanobactins patellins 2 and 3 (*truE1* gene product) is shown for comparison on the gray background. Functional parts of the peptide including leader (yellow), two cores (red), and recognition sequences of peptidases (RS1 and RS2) are shown (Gu et al., 2018). **(C)** Sequences of cassette-containing precursor peptides of flavazolicins showing the conserved positions in two leader sequences. Conserved positions are shown with green dashed lines, synonymous substitutions with yellow dashed lines. **(D)** A possible scenario in the evolution of cassette-containing peptides. See main text for the explanations.

The last group of putative new LAP BGCs was identified during the analysis of precursor peptides predicted with RiPPER (Santos-Aberturas et al., 2019). The precursor peptide identified in the genome of flavobacterium *Algibacter aquaticus* SK-16 (a singlet and therefore not shown in Figure 6A; Figure 8A) appears to have resulted from a duplication of a standard leader-core ancestral precursor gene (Figure 8B). As a result, in a single ORF, there are two putative core sequences rich in Ser and Cys residues separated by an “internal” leader (another leader is N-terminally located) (Figure 8C). A similar cassette-like arrangement of core peptides has been described for several different groups of RiPPs including cyanobactins (Gu et al., 2018), thiovarsolines (Santos-Aberturas et al., 2019), orbitides (Shim et al., 2015), and dikaritins (Ding et al., 2016); but in all these cases, precursors are composed of a single leader, followed by several core peptides, interspersed by signal sequences required for the cleavage of each core at C- and N-termini by dedicated peptidases (Figure 8B shows, as an example, the sequence of TruE1 — the precursor of patellins 2 and 3, representatives of cyanobactins).

A BLAST search for similar BGCs resulted in identifying six additional BGCs, that share the same set of modification enzymes (Figure 8A). The first three originate from the genomes of *Flavobacteriaceae* closely related to *Algibacter*, while three others were found in the genomes of *Gammaproteobacteria*. Interestingly, only two of these clusters contained a fused precursor peptide gene, while the rest had a set of one to three separate ORFs encoding non-fused precursor peptides (Figure 8B). These different genome arrangements from the closely related species provide a glimpse on how the genes of cassette-containing peptides may originate from an independent single short ORF through gene duplication [Figure 8D(1)], fusion [Figure 8D(2)], and a subsequent reduction of the role of the internal leader to that of a recognition sequence of proteases [Figure 8D(4)]. Further multiplication of cassette-containing precursor genes may lead to arrangements found in several cyanobactin clusters [Figure 8D(5); Gu et al., 2018].

Strikingly, only the BGCs with fused precursors contain an additional gene, which is a predicted protease (Figure 8A, orange). This enzyme may be involved in the processing required to produce individual modified core parts. The acquisition of an additional protease gene may be the step that follows the fusion of two independent ORFs in the course of cassette-containing BGC evolution [Figure 8D(3)]. We named the products of this family of BGCs *flavazolicins*. Characterizing the products encoded in these BGCs and establishing the details of their biosynthesis and function appears to be an exciting direction of future work.

## Concluding Remarks

Although the number of the known subclasses and unique representatives of RiPPs increases each year, a remarkable proportion of publications devoted to novel compounds provides information only about the structure and sometimes evaluates the bioactivity of a modified peptide. Researchers focusing on RiPP clusters as a source of unprecedented enzymatic activities rarely proceed toward establishing the mode of action of the target compound and are even less likely to establish its physiological or ecological role. Addressing these questions is a challenging task, which partially explains the lack of detailed information about the precise mechanisms of action for many groups of RiPPs, including some that are known and have been studied for decades (e.g., bottromycin, McB). We hope that the upcoming years will provide more structural insights not only on the enzymology of RiPP modification widely studied now, but also on the principles the already known and novel compounds act by.

In many cases, the analysis of genomic information was a starting point for further successful discoveries of a novel RiPP, facilitating the prediction of the BGC product based on the sequences of precursor peptides and modification enzymes. Through genome mining, future studies will not only result in the discovery of new compounds but will also allow systemization of our knowledge about RiPP genomic landscape and a better understanding of RiPP clusters’ evolutionary relations.

## Methods

### Search for YcaO Containing BGCs, Filtration, and Annotation

146,381 bacterial genomes were downloaded from RefSeq (O’Leary et al., 2016) database on 27 March 2019. To obtain all YcaO domain-containing proteins we searched the database with profile HMMs (TIGR03549, TIGR03604, and PF02624) from public databases using hmmer package^1^. We clustered resulting hits with mmseqs2 (Mirdita et al., 2019) (90% identity; 90% coverage) to remove duplicates and redundant highly similar sequences from organisms, which genome sequences are overrepresented in the database.

The genomic regions of 12.5 kbp to each side of the identified unique YcaO protein-coding genes were annotated with RODEO (Tietz et al., 2017) using Pfam 32.0 and TIGRFAMs 15.0 databases. For further analysis, we selected genomic regions according to several rules. First, we collected regions that encode proteins containing E1-like (PF00881, TIGR03603, TIGR04424) or ThiF-like (PF00899, TIGR02354, TIGR02356, TIGR03693, TIGR03736, TIGR03882) domains. Initial search was very sensitive and false positive results were obtained. Thus, we removed predicted YcaO proteins that were not annotated with TIGR03549, TIGR03604, or PF02624 domains in the RODEO output. In order to exclude thiopeptides, studied comprehensively in several other works, we removed genomic regions containing genes of lantipeptide dehydratase (PF14028, PF04738, TIGR03897, PF05147). Putative precursor peptides were predicted with RiPPER (Santos-Aberturas et al., 2019). For each BGC, the best predicted precursor peptide was selected as the one bearing the highest number of cyclizable residues (Ser, Thr, Cys residues) within the C-terminal half.

Using a custom script (available on http://github.com/bikdm12/RODEO2antiSMASH) we converted RODEO output to genbank files imitating antiSMASH (Blin et al., 2019) output. The script adds a feature “cluster” with information about the class of the product. The coordinates of this feature are boundaries of the group of genes located on the same strand not farther than 100 bps from each other and containing YcaO protein. Also, genes that may be related to azol(in)e-containing RiPP biosynthesis (for the list of domains see Supplementary Table S3) were marked as biosynthetic. These files were then used to build a sequence similarity network with BiG-SCAPE (Navarro-Muñoz et al., 2020) subsequently visualized with Cytoscape (Shannon et al., 2003).

## Author Contributions

DT and DB performed the bioinformatic search and interpreted its results. DT prepared the figures. All authors wrote the manuscript.

## Conflict of Interest

The authors declare that the research was conducted in the absence of any commercial or financial relationships that could be construed as a potential conflict of interest.

## Footnotes

1 hmmer.org

## Supplementary Material

TABLE S1 Manually curated set of previously characterized YcaO-containing BGCs (including bioinformatically predicted, but not validated experimentally).

TABLE S2 List of YcaO-containing BGCs with E1-like or ThiF-like YcaO partner proteins identified in this study (exluding clusters encoding lantipeptide dehydratases).

TABLE S3 List of Pfam and TIGRFAMs profile HMMs used to mark biosynthetic genes.
